# Supplementary material for: Content-rich biological network constructed by mining PubMed abstracts
Source: BMC Bioinformatics. 2004 Oct 8;5:147. doi: 10.1186/1471-2105-5-147 (PMC528731; doi:10.1186/1471-2105-5-147)
Supplement: Additional File 5 — The original Chilibot query results of the term "long-term potentiation (LTP)" and 22 other terms, limiting the latest references analyzed to the years 1990, 1995, 2000, and 2004. [file 1471-2105-5-147-S5.bz2 › chilibotAdditionalFile5/ltp2000/html/NMDA_AMPA.html]

 


 **NMDA** and **AMPA** 
  
Found 2355 abstracts in PubMed,  **30 abstracts were retrieved and analyzed**.  


---

 Search Google  |
 PDF files only 
|  EDU domain only 

---

**Interactive relationship** (e.g. stimulation, inhibition, etc)

- Here we show that activation of  **NMDA**  receptors alone can trigger  **AMPA**  receptor endocytosis through calcium influx and activation of the calcium dependent protein phosphatase calcineurin.  Ref: 11100150 Nat Neurosci, 2000
- These results demonstrate that although multiple signaling pathways can induce  **AMPA**  receptor internalization,  **NMDA**  receptor activation enhances  **AMPA**  receptor endocytosis via a signaling mechanism required for the induction of LTD.  Ref: 11100150 Nat Neurosci, 2000
- The endocytosis of  **AMPA**  receptors is thought to be important in the expression of long term depression LTD triggered by  **NMDA**  receptor activation.  Ref: 11100150 Nat Neurosci, 2000
- Our findings suggest that the anticonvulsant properties of riluzole depend upon its interaction with neurotransmission mediated by both the glycine  **NMDA**  and the  **AMPA**  kainate receptor complex.  Ref: 11070180 Eur J Pharmacol, 2000
- The present data reveal that  **NMDA**  receptors are co localized with variants of  **AMPA**  receptors in a large proportion of vestibular nuclear neurons.  Ref: 11082490 Brain Res, 2000
- We find that glutamate input is necessary for clustering the  **AMPA**  type glutamate receptor but not for clustering the  **NMDA**  receptor or the associated PSD 95 family scaffold in GABAergic cells.  Ref: 11069941 J Neurosci, 2000
- Type 3 cells had both  **NMDA**  and alpha amino 3 hydroxy 5 methyl 4 isoxazolepropionic acid  **AMPA**  current although the  **NMDA**  component could be isolated at low stimulus intensity  **NMDA**  threshold< ;  **AMPA**  threshold.  Ref: 11113509 Brain Res Dev Brain Res, 2000
- Furthermore,  **AMPA**  receptors were found to be more sensitive to fullerenols than  **NMDA**  and KA receptors.  Ref: 11070504 J Neurosci Res, 2000
- The data obtained suggest that hydrophobic and nucleophilic components of the binding site are located close to each other in the channels of the  **NMDA**  receptor type but are separated by approximately 10 A in the  **AMPA**  receptor channel.  Ref: 11081219 Ross Fiziol Zh Im I M Sechenova, 2000
- The induction of graded burst discharges in magnesium free conditions and the expression of burst discharges evoked in normal bathing medium with subsequent disinhibition were both blocked by DL 2 amino 4 phosphonovaleric acid APV and were therefore  **NMDA**  receptor dependent, in contrast to long term potentiation LTP in the perforant path, which is induced by  **NMDA**  receptors and is also expressed by alpha amino 3 hydroxy 5 methyl 4 isoxazoleproprionate  **AMPA**  receptors.  Ref: 11110816 J Neurophysiol, 2000
- The  **AMPA**  KA antagonist DNQX was more effective, although not additive in preserving retinal ganglion cells after optic nerve crush than the  **NMDA**  antagonist MK801.  Ref: 11095632 Invest Ophthalmol Vis Sci, 2000

**Parallel relationship** (e.g. studied together, co-existance, homology, etc.)

- Expression of  **NMDA** ,  **AMPA**  and GABA A receptor subunit mRNAs in the rat auditory brainstem.  Ref: 11077190 Hear Res, 2000
- Lubeluzole selectively increased gamma amino butyric acid A GABA A receptor binding but had no significant and or consistent effects on N methyl D aspartate  **NMDA** , alpha amino 3 hydroxy 5 methylisoxazole 4 propionic acid  **AMPA** , and kainate receptors.  Ref: 11108997 Neurosci Lett, 2000
- The present study was carried out to investigate the effects of lead exposure on non  **NMDA**  alpha amino 3 hydroxy 5 methyl 4 isoxazole propionic acid,  **AMPA**  kainate receptors of rat hippocampus.  Ref: 11129499 Pharmacol Toxicol, 2000
- Expression of mRNAs encoding ionotropic glutamate receptor subunits such as alpha amino 3 hydroxy 5 methyl 4 isoxazole  **AMPA**  and N methyl D aspartate  **NMDA** , and gamma aminobutyric acid type A GABA A receptor subunits was assessed by in situ hybridization in the dorsal DCN and the ventral cochlear nucleus VCN and in the central nucleus of the inferior colliculus CNIC.  Ref: 11077190 Hear Res, 2000
- We characterised the excitatory and inhibitory synaptic currents of MSO neurones in 3 to 14 day old rats using whole cell patch clamp methods in a brain slice preparation.A dual component EPSC was mediated by  **AMPA**  and  **NMDA**  receptors.  Ref: 11118498 J Physiol, 2000
- We found an age related decline in the number of  **NMDA**  receptors in the CA1, CA3 and subicular cell regions of the hippocampus... and an age related decline in the number of  **AMPA**  receptors in the FD hilar region, but not in the CA fields.  Ref: 11121523 Brain Res, 2000
- Seven rat brain neurotransmitter receptors were selected to study because of their reported roles in cognitive function receptors for nicotine, muscarine,  **AMPA**  kainate, N methyl D aspartate  **NMDA** , gamma aminobutyric acid, and dopamine 1 and 2.  Ref: 11076700 Toxicol Appl Pharmacol, 2000
- After 15 days of daily unilateral ICES, the expressions of NR1, NR2b and NR2c subunits of  **NMDA**  receptor, that of GluRA, B, C, D flop isoforms of  **AMPA**  receptor.  Ref: 11077190 Hear Res, 2000
- Semi quantitative RT PCR measurements of neuronal TGFbeta2 mRNA showed that  **NMDA**  or  **AMPA**  kainate receptor stimulation reduced TGFbeta2 mRNA levels.  Ref: 11122374 Eur J Neurosci, 2000
- The effect of N methyl D aspartate  **NMDA**  and 2 aminomethyl phenylacetic acid kainate  **AMPA**  kainate glutamate receptors on dentate cell proliferation and hippocampal synapsin I induction was examined after global ischemia.  Ref: 11129783 J Cereb Blood Flow Metab, 2000
- It is based on the assumption that, in diverse central nervous system structures, long term potentiation depression LTP LTD of excitatory transmission depression potentiation of inhibitory transmission, LTDi LTPi is the result of an increasing decreasing the number of phosphorylated  **AMPA**  and  **NMDA**  GABA A receptors.  Ref: 11084240 BiosystemsBiosystems, 2000
- Groups of animals were treated with an  **NMDA**  antagonist, an  **AMPA**  KA antagonist, or both.  Ref: 11095632 Invest Ophthalmol Vis Sci, 2000
- Furthermore, cobalt accumulations induced by  **NMDA**  and  **AMPA**  in the lead exposed rats decreased significantly than those in the controls.  Ref: 11129499 Pharmacol Toxicol, 2000
- The decline in the number of  **NMDA**  or  **AMPA**  receptors that occurs with aging was not a continuous or homogeneous process.  Ref: 11121523 Brain Res, 2000
- After pre treatment with naloxone, d methadone was unable to inhibit nociception in the Randall Selitto model, the  **NMDA**  or  **AMPA**  evoked neuronal activity or the motoneurone wind up.  Ref: 11108995 Neurosci Lett, 2000
- Expression of  **NMDA** ,  **AMPA**  and GABA A receptor subunit mRNAs in the rat auditory brainstem.  Ref: 11077189 Hear Res, 2000
- Regional changes in the hippocampal density of  **AMPA**  and  **NMDA**  receptors across the lifespan of the rat.  Ref: 11121523 Brain Res, 2000
- **NMDA**  and  **AMPA**  kainate glutamate receptors modulate dentate neurogenesis and CA3 synapsin I in normal and ischemic hippocampus.  Ref: 11129783 J Cereb Blood Flow Metab, 2000
- Type 4 cells had both  **AMPA**  and  **NMDA**  currents with  **NMDA**  threshold> = **AMPA**  threshold.  Ref: 11113509 Brain Res Dev Brain Res, 2000
- calcium permeable  **AMPA**  kainate receptors in organotypic slice cultures were evaluated by using cobalt uptake, a histochemical method that identifies cells expressing calcium permeable non  **NMDA**  receptors.  Ref: 11129499 Pharmacol Toxicol, 2000
- Known genes that were upregulated in waking and sleep deprivation can be grouped into the following categories immediate early genes transcription factors, genes related to energy metabolism, growth factors adhesion molecules, chaperones heat shock proteins, vesicle and synapse related genes, neurotransmitter hormone receptors and beta, GABA receptor beta, glutamate  **NMDA**  receptor 2A, glutamate  **AMPA**  receptor GluR2 and GluR3, nicotinic acetylcholine receptor beta, thyroid hormone receptor TRbeta, neurotransmitter transporters Cl transporter NTT4 Rxt1, enzymes, and a miscellaneous group.  Ref: 11102586 Brain Res, 2000
- Combining two GABA mimetic drugs or combining an  **AMPA**  antagonist with an  **NMDA**  antagonist may enhance efficacy, but tolerability is sometimes reduced.  Ref: 11077449 Epilepsia, 2000
- The current study dissected the fascia dentata FD and hilar region from the CA and subicular cell fields of the rat and conducted in vitro determinations of the number of binding sites for N methyl D aspartate  **NMDA**  and alpha amino 3 hydroxy 5 methyl 4 isoxazole  **AMPA**  glutamate receptors across the lifespan.  Ref: 11121523 Brain Res, 2000
- We conclude that the MSO receives excitatory inputs mediated by  **AMPA**  and  **NMDA**  receptors and a strong glycinergic IPSC which has a significant contribution from GABAA receptors in neonatal rats.  Ref: 11118498 J Physiol, 2000
- Neither spontaneous efflux nor evoked D ASP release were affected by  **NMDA** ,  **AMPA**  or group I metabotropic glutamate receptor mGluR antagonists.  Ref: 11099785 Neurochem Int, 2001
- We determined the density of binding of 3H glutamate or 3H  **AMPA**  to  **NMDA**  or  **AMPA**  receptor sites, respectively.  Ref: 11121523 Brain Res, 2000
- d Methadone inhibited the activity of spinal dorsal horn neurones evoked by both iontophoretic  **NMDA**  and R, S alpha amino 3 hydroxy 5 methyl 4 isoxazole propionic acid  **AMPA** , ED 50 5.7 and 8.2 mg kg i.v.  Ref: 11108995 Neurosci Lett, 2000
- Ganglion cell loss after optic nerve crush mediated through  **AMPA**  kainate and  **NMDA**  receptors.  Ref: 11095632 Invest Ophthalmol Vis Sci, 2000
- By analogy with silent synapsesi.e. synapses that contain only  **NMDA**  receptors, Type 2 cells are candidate silent cells that may undergo activity dependent acquisition of functional fast conducting  **AMPA**  receptors with maturation.  Ref: 11113509 Brain Res Dev Brain Res, 2000
- The topography of the channel binding site in glutamate receptors  **AMPA**  and  **NMDA**  types of rat brain neurons, receptors of molluscan neurons and insect muscle, and in two subtypes of nicotinic cholinoreceptors in frog muscle and cat sympathetic ganglion, has been investigated by comparison of the blocking effects of mono and dicationic derivatives of adamantane and phenylcyclohexyl.  Ref: 11081219 Ross Fiziol Zh Im I M Sechenova, 2000
- We are interested in studying the co localization of  **NMDA**  glutamate receptor subunits and  **AMPA**  glutamate receptor subunits in individual neurons of the rat vestibular nuclei.  Ref: 11082490 Brain Res, 2000
- Co expression of  **NMDA**  receptor subunits and  **AMPA**  receptor subunits was demonstrated by double labeling of NR1 GluR1, NR1 GluR2 3, NR1 GluR4 and NR2A B GluR2 in individual vestibular nuclear neurons.  Ref: 11082490 Brain Res, 2000
- **NMDA**  and  **AMPA**  kainate receptors are involved in the anticonvulsant activity of riluzole in DBA 2 mice.  Ref: 11070180 Eur J Pharmacol, 2000
- The expression of mRNAs encoding N methyl D aspartate  **NMDA** , alpha amino 3 hydroxy 5 methyl 4 isoxazole  **AMPA**  and gamma aminobutyric acid type A GABA A receptor subunits was then studied by in situ hybridization in the dorsal and ventral cochlear nucleus and in the central nucleus of the inferior colliculus CNIC.  Ref: 11077189 Hear Res, 2000
- In the presence of ionotropic glutamate receptor iGluR agonists, including N methyl D aspartic  **NMDA** , DL alpha amino 3 hydroxy 5 methylisoxasole 4 propionic  **AMPA**  and kainic acids KA, at concentrations maximally effective in displacing each receptor binding, the agonists for group I mGluR subtype 1 aminocyclopentane trans dicarboxylic acid trans ACPD and S dihydroxyphenylglycine S DHPG more potently displaced 3H QA binding in a concentration dependent manner than their absence.  Ref: 11099787 Neurochem Int, 2001
- These results suggest that cross modulation between  **NMDA**  receptors and  **AMPA**  receptors may occur in individual neurons of the vestibular nuclei during glutamate mediated excitatory neurotransmission and may in turn contribute to synaptic plasticity within the vestibular nuclei.  Ref: 11082490 Brain Res, 2000
- Long term exposure 48 h of cortical neurons to selective antagonists of N methyl D aspartate  **NMDA**  and alpha amino 3 hydroxy 5 methyl 4 isoxazolepropionic acid  **AMPA**  kainate receptors markedly increased TGFbeta2 levels in the culture medium.  Ref: 11122374 Eur J Neurosci, 2000
- Although most investigators have focused on blockade of the N methyl D aspartate  **NMDA**  subtype of glutamate receptor, we have chosen to evaluate the efficacy of blockade of the  **AMPA**  kainate KA receptor in this experimental paradigm.  Ref: 11095632 Invest Ophthalmol Vis Sci, 2000
